# Detection of breast cancer precursor lesions by autofluorescence ductoscopy

**DOI:** 10.1007/s12282-020-01136-6

**Published:** 2020-07-28

**Authors:** Laurien Waaijer, Mando D. Filipe, Janine Simons, Carmen C. van der Pol, Tjeerd de Boorder, Paul J. van Diest, Arjen Joost Witkamp

**Affiliations:** 1 Department of Surgery, University Medical Center Utrecht, Heidelberglaan 100, 3584 CX Utrecht, The Netherlands; 2 Department of Medical Technology and Clinical Physics, University Medical Center Utrecht, Heidelberglaan 100, 3584 CX Utrecht, The Netherlands; 3 Department of Pathology, University Medical Center Utrecht, Heidelberglaan 100, 3584 CX Utrecht, The Netherlands

**Keywords:** Ductoscopy, Breast cancer, Diagnosis

## Abstract

**Purpose:**

Autofluorescence is an image enhancement technique used for the detection of cancer precursor lesions in pulmonary and gastrointestinal endoscopy. This study evaluated the feasibility of addition of autofluorescence to ductoscopy for the detection of intraductal breast cancer precursor lesions.

**Methods:**

An autofluorescence imaging system, producing real-time computed images combining fluorescence intensities, was coupled to a conventional white light ductoscopy system. Prior to surgery, ductoscopy with white light and autofluorescence was evaluated under general anaesthesia in women scheduled for therapeutic or prophylactic mastectomy. Endoscopic findings in both modes were compared, marked and correlated with histology of the surgical specimen.

**Results:**

Four breast cancer patients and five high-risk women, with a median age of 47 years (range 23–62) were included. In autofluorescence mode, two intraductal lesions were seen in two breast cancer patients, which had an increase in the red-to-green fluorescence intensity compared with the surrounding tissue. One lesion had initially been missed by white light ductoscopy but was clearly visible in subsequent autofluorescence mode. One endoscopic finding was classified as suspicious by white light, but was negative in autofluorescence mode and showed normal histology.

**Conclusions:**

This study demonstrates for the first time the in vivo feasibility of autofluorescence ductoscopy to detect pathologically confirmed breast cancer precursor lesions in both breast cancer patients and high-risk women that were occult under white light.

## Introduction

Breast cancer is, with 523,000 new cases a year, the most common type of cancer and accounts for 138,000 deaths a year in Europe [1]. Hereditary breast cancer accounts for up to 5–10% of all breast cancers with two high-penetrance genes (*BRCA1* and *BRCA2*) responsible for about 16% of the familial risk of breast cancers, associated with a 60–80% lifetime risk of developing breast cancer [2–5]. Currently, the ultimate prevention in these women is bilateral prophylactic mastectomy [6]. Consequently, this means that 20–40% of patients undergo mastectomies without signs of malignancy. Unfortunately, mastectomies are accompanied by complications along with serious cosmetic and psychological consequences [7, 8].

Most breast cancers are thought to arise from the ductal epithelium [9, 10]. An appealing approach would be to target breast cancer precursors originating from the epithelial lining of the breast ducts through ductoscopy. This is a minimally invasive microendoscopic technique, which makes real-time visualisation of the milk ducts of the breast possible. Ductoscopy is currently performed under local anaesthesia at the outpatient clinic, and is currently mainly applied as an additional diagnostic tool in the work-up of women suffering from pathological nipple discharge (PND) without suspicious radiological findings [11–18]. Different studies show that ductoscopy can accurately detect intraductal lesions causing PND before or during duct excision [19–23]. The role of ductoscopy in breast-cancer screening and breast conservation surgery has yet to be fully defined [24], but the first studies using autofluorescence in ductoscopy indicated the feasibility and the possibility to detect malignant lesions [25, 26]. The former study was an ex vivo study, the latter a technical in vivo feasibility study in three patients not aimed at detecting lesions, and without taking material for pathological evaluation. (Pre)malignant epithelial lesions show an aberrant pattern under fluorescent light by which they become detectable, as is already known from the airways, larynx and colon [27–29]. However, the breast ductal system had not been evaluated before by autofluorescence to detect pathologically confirmed precursor lesions.

From a prospective feasibility study in patients affected by breast cancer and in women with a known mutation in *BRCA1* or *BRCA2*, we report for the first time the in vivo feasibility of autofluorescence to detect (white light occult) breast cancer precursor lesions by autofluorescence ductoscopy, confirmed by histology of the subsequently performed mastectomy.

## Methods

### Patients

A prospective observational phase II cohort study was conducted in adult women who underwent either therapeutic or prophylactic mastectomy in the University Medical Center Utrecht, The Netherlands, between October 2014 and May 2015.

Two cohorts were included. The therapeutic cohort consisted of 4 female patients undergoing a mastectomy for recently diagnosed invasive breast cancer or ductal carcinoma in situ (DCIS). The prophylactic cohort consisted of 5 women undergoing prophylactic mastectomy for increased risk of breast cancer. The first cohort hypothetically carries multiple precursor lesions and serves as a reference for the autofluorescence ductoscopy technique; the second is the index population that will provide information about the diagnostic value of this technique in high-risk patients.

Patients with previous surgery or radiotherapy of the breast were excluded. This study was approved by the Institutional Review Board of the UMC Utrecht. All patients provided written informed consent.

### White light and autofluorescence ductoscopy

Study procedures were conducted immediately prior to mastectomy, and were all performed under sterile conditions and under general anaesthesia in the operation room.

First, a saline solution was injected around the nipple to cause thrust and thereby exposing the orifices of the milk ducts. Salivary duct probes (Karl Storz, Tuttlingen, Germany) size 0000 to 1 and an obturator (Polydiagnost GmbH, Pfaffenhofen, Germany) were used for dilatation of one of the duct orifices in the nipple. The introduction port (SoLex-Nipple-Expander^®^, Polydiagnost) or a custom-made introduction port compatible with the Storz endoscope was placed into the duct orifice through which the ductoscope was introduced. A 0.55 mm optic (LaDuScope T-flex, Polydiagnost) was inserted in a 1.15 mm outer diameter Polyshaft (PD-DS-1015, Polydiagnost) or a Storz miniature endoscope (Erlangen, Karl Storz) with incorporated fiberoptic light transmission and an outer diameter of 1.1 mm, was used. Both devices are semiflexible and have a separate irrigation channel for saline-infusion, and a working channel (diameter 0.45 mm).

The ductoscope was coupled via a custom-made eyepiece to an autofluorescence endoscopic imaging system (OncoLIFE^®^, Xillix Technologies Corporation, British Columbia, Canada, now Pinpoint^®^, Novadaq Technologies Corporation, Ontario, Canada). Method of operation was described previously by Douplik et al. [25]. Briefly, white-light and autofluorescence images were recorded using 6.3-mW broadband light and 5.3-mW blue band (390 to 450 nm), respectively. In other organs, premalignant tissues have a reduced green autofluorescence relative to normal tissues when excited by blue light; normal tissue appears as cyan, while abnormal tissue is shown red coloured [28, 30]. In autofluorescence mode, the central 16–12 pixels are averaged over four frames and continuously displayed as a numerical color value (NCV). The higher the NCV, the lower the autofluorescence intensity, which has been associated with neoplasia [31].

First, standard (white-light mode) ductoscopy was performed. Whenever suspicious findings were encountered, we switched to autofluorescence mode by a hand switch or foot pedal. When no suspicious findings were encountered under white light, the entire duct was examined by autofluorescence ductoscopy.

White light findings were classified as normal (no visual abnormality) or abnormal (irregularity of the ductal lining such as redness, hypervascularity, swelling, thickening, as well as nodular or polypoid lesions). In autofluorescence, a green colour was classified as normal, while areas showing red colour with decreased autofluorescence were classified as abnormal. In autofluorescence mode NCV values were continuously monitored.

In the first seven patients, the ductoscopy procedure was performed via one single duct orifice, to limit operation time. In the 8th case, multiple ducts were examined. In the therapeutic cohort ductoscopic exploration was performed in the breast quadrant containing malignancy to encounter the previously established lesion. In the prophylactic cohort the duct orifice that was easiest to cannulate was chosen and only one breast was examined. In case of an abnormality in the studied duct, 1–2 ml colour marker (sterile Black Eye Endoscopic marker™, The Standard, Korea) was placed through the working channel of the ductoscope after removal of the optic, to facilitate precise histological correlation. Distance of the lesion to the nipple was also recorded. When no abnormalities were found, the most extensively examined duct was marked. Images of ductal abnormalities were recorded. All ductoscopy procedures were performed by the same physicians (AW, CP).

Following the ductoscopy procedure a conventional (therapeutic or prophylactic) mastectomy was performed.

### Pathology

Mastectomy specimens were submitted fresh to pathology, where the margins of the specimen were inked with non-black colours to avoid interference with the intraductal dye-mark. The specimen was sliced in 5-mm slices, and scrutinized for the marked area and macroscopic lesions. At the level of the colour marker, the specimen was totally embedded at a transversal plane to acquire a trans sectional view of the duct. All tissue was formalin fixed and used for routine histological evaluation using conventional haematoxylin–eosin (HE) staining. At the level of the tumour the specimen was embedded according to standard procedure.

All intraductal abnormities were described. Assessment of the surgical specimens was performed by one dedicated breast pathologist (PD) blinded to the endoscopic results.

### Follow up

Decisions regarding postoperative treatment with adjuvant chemotherapy or hormonal therapy were made according to usual protocols based on patients’ risk category and based on the tumour characteristics, size and stage.

### Evaluation and analysis

Primary endpoint was the technical feasibility, determined by cannulation success and findings of intraductal abnormalities. Endoscopic findings in white light, autofluorescence and NCVs were correlated to final histology of the surgical specimen. Differences in endoscopic findings under white light and autofluorescence ductoscopy were described.

## Results

Table 1 shows the patient and imaging characteristics of the 9 included patients. Duct cannulation and subsequent ductoscopic exploration were successful in 8 of 9 (89%) women. In one patient undergoing prophylactic mastectomy cannulation failed due to narrow duct orifices.

**Table 1:** Ductoscopic, radiologic, and patient characteristics of the two cohorts

	Therapeutic mastectomy, *n* = 5	Prophylactic bilateral mastectomy, *n* = 4
Mean age (range), years	50 (range 29–62)	44 (range 23–61)
Spontaneous nipple discharge	0	0
Ductoscopy side, N*		
Left	3	3
Right	2	1

*In bilateral prophylactic mastectomy, the ductoscopy was performed unilateral, in the most accessible duct orifice for cannulation. Reported here is the ductoscopy side where cannulation was successful

Ductoscopic examination time, from cannulation to termination of the procedure ranged from 15 to 45 min (mean, 28.3 min). This is in line with conventional ductoscopy, additional time for autofluorescence examination ranged from 5 to 8 min.

### Breast cancer patients

**Fig. 1:**
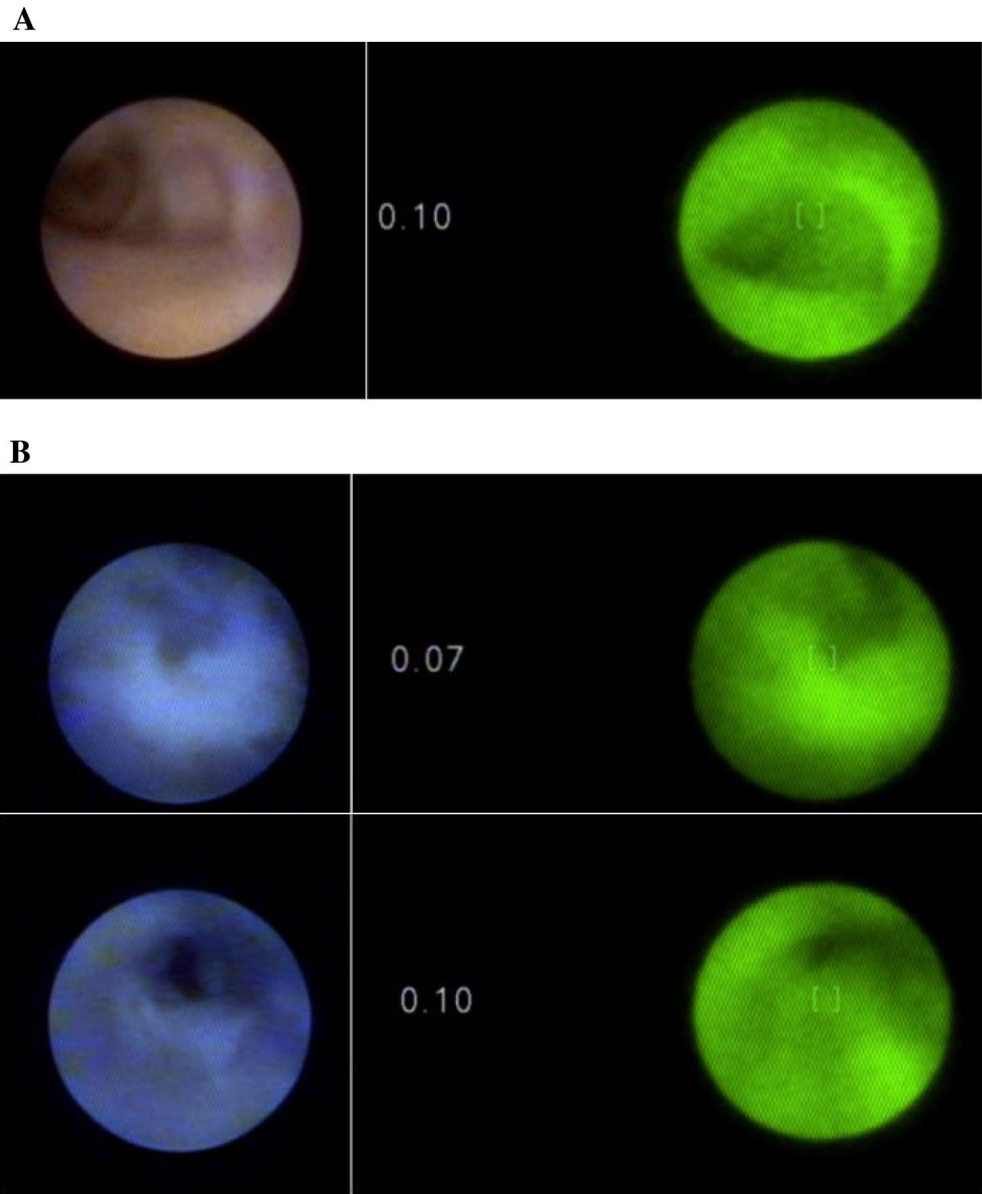
**a** Left: normal-appearing duct in white light. Right: same duct in autofluorescence mode, showing green colour and corresponding low NCV. **b** Upper and lower left: intraductal abnormalities in white light. Upper and lower right: showing the same abnormalities in autofluorescence mode, with green colour and corresponding low NCV

In four of five breast cancer patients solitary (*n* = 2) or multiple (*n* = 2) intraductal abnormalities were visualised in with light and/or autofluorescence mode. Table 2 shows the characteristics of the lesions found. Normal appearing ducts in white light coloured green in autofluorescence mode, corresponding with low NCVs (Fig. 1a). In patient 1, a deposition in the lining of a duct was seen with autofluorescence mode, but not by white light mode, at around 4–5 cm from the nipple. In this patient, the first cannulation attempt was unsuccessful. The pathology report showed that DCIS was also found in the nipple area.

**Table 2:** Endoscopic findings per patient

Age	Study ID	Ducts cannulated, *N*	Cohort	Histology (excision specimen)	White light	Autofluorescence	NCV max (autofluorescence mode)	Histology marked duct
62	1	1	Therapeutic	DCIS	+ Friable intraductal duct lining depositions or debris. Distance to nipple: 4–5 cm	–	0.10	–
23	2	1	Prophylactic BRCA2	No	–	–	0.25	–
61	3	1	Prophylactic BRCA1	(Pre)malignancy No	–	–	0.13	–
47	4	1	Therapeutic	(Pre)malignancy multicentric IDC and DCIS	+ Red-coloured flat lesion of ductal lining. Distance to nipple: 7 cm	+	0.43	+
41	5	0	Prophylactic	No (pre)malignancy	NA	NA	NA	Ductal hyperplasia usual type
62	6	1	BRCA1 therapeutic	ILC and LCIS	–	–	0.18	–
29	7	1	Therapeutic	Multicentric DCIS and focal micro-invasive carcinoma	A. − Intraductal polypoid lesion initially missed by white light ductoscopy. Distance to nipple: 4 cm B. + Friable duct lining depositions. Distance to nipple: 4 cm	A. + B. −	A. 1.53 B. 0.53	Ductal hyperplasia usual type and apocrine metaplasia
52	8	1	Prophylactic BRCA1	No (pre)malignancy	–	–	0.20	–
50	9	1	Therapeutic	DCIS	–	+. Duct lining deposition. Distance to nipple: 6 cm	0.30–0.40	–

NA = not applicable, due to unsuccessful cannulation no ductoscopy could be performed

*DCIS* ductal carcinoma in situ, *IDC* invasive ductal carcinoma, *ILC* invasive lobular carcinoma, *LCIS* lobular carcinoma in situ

In a second breast cancer patient (patient 4), three friable intraductal abnormalities (debris) were visualised in white light mode at 7 cm from the nipple. In autofluorescence mode these abnormalities where green-coloured with NCVs < 0.10 (Fig. 1b). Histology of the examined duct showed the intraductal marker, confirming the correct localization. Histology showed no abnormalities.

**Fig. 2:**
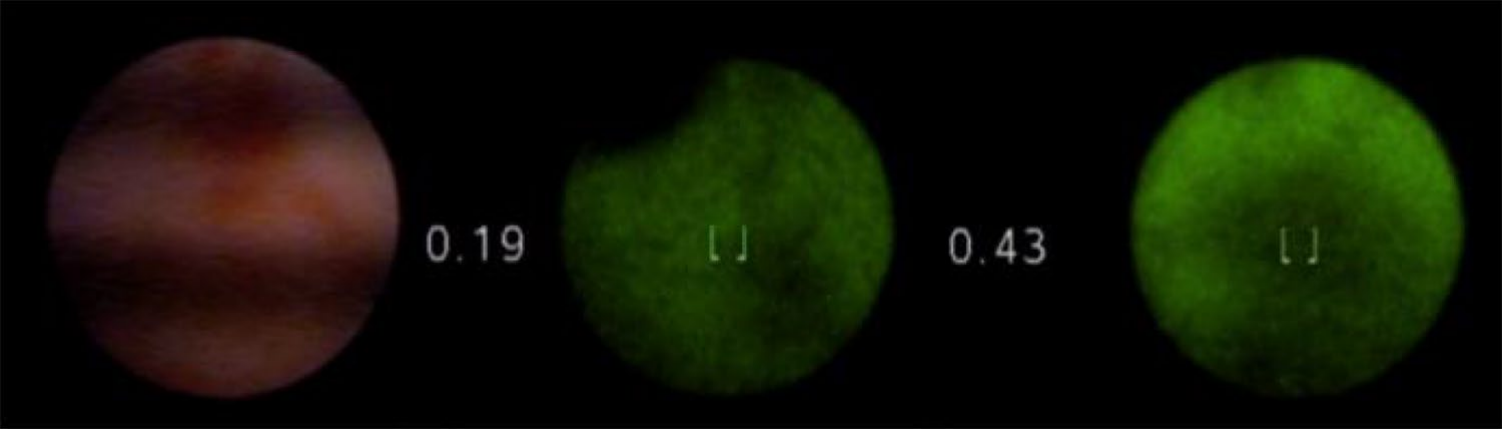
Flat epithelial lesion in white light. On middle and right: showing the same lesion in autofluorescence mode, showing a red-coloured contrast with the green ductal lining and showing increased NCVs (0.43)

In patient 6, a haemorrhagic, red-coloured epithelial lesion was seen under white light, showing normal green colour in autofluorescence mode with maximum NCVs of 0.43 (Fig. 2). Histology of the duct showed the intraductal marker, confirming the correct localization, with ductal epithelial hyperplasia and epithelial damage.

**Fig. 3:**
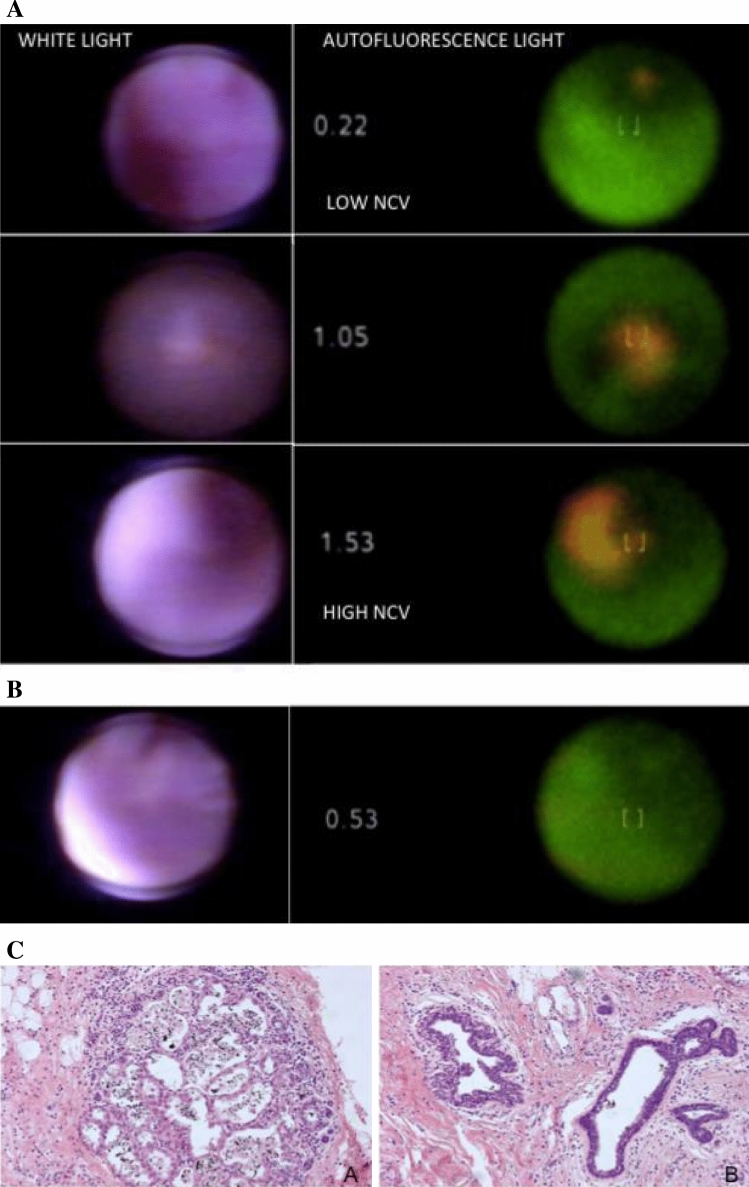
**a** Ductoscopic images taken immediately prior to therapeutic mastectomy for multicentric DCIS grade 3. White light ductoscopy (left). Upper: showing an intraductal puffy aspect of the duct wall with ductal asymmetry (oval shape). Middle: intraductal polypoid lesion, not clearly identifiable from the duct wall. Lower: close up of the lesion. Autofluorescence ductoscopy (right). Upper: focusing on the normal, green coloured, duct wall. NCV shows corresponding low values (0.22). Middle: focusing on the intraductal red coloured intraductal abnormality, NCV shows corresponding high NCV values (1.05). Lower: Close-up of the same lesion, high NCVs (1.53). **b** Endoscopic view in the same ductal system. Autofluorescence and white light image of the high NCVs on a small area of duct lining. Friable lesions seen in white light, showing no clear red-colour but increased NCVs. **c** Histology of the examined ductal system of figures **a** and **b** show the intraductal black dye marking and apocrine metaplasia and ductal hyperplasia of the usual type. Since the dye-marker colours the complete examined ductal tree, precise correlation of the two separate intraductal abnormalities shown in **a** and **b** is not possible

In patient 7, an intraductal polypoid lesion appearing as irregular protrusion into the ductal lumen, with colours similar to the surrounding ductal tissue, was initially missed by white light ductoscopy at 4 cm from the nipple. In autofluorescence mode, the same lesion was displayed as a red-coloured intraductal polypoid lesion clearly contrasting the surrounding ductal tissue, with maximum NCVs of 1.53 (Fig. 3a). In the same ductal tree, another abnormality was visualised (Fig. 3b); friable ductal wall adhesions seen in white light, showed no clear red-colour in autofluorescence mode but did show maximum NCVs of 0.53. Histology of the examined ductal system showed the intraductal black dye-marking with both apocrine metaplasia and ductal hyperplasia (Fig. 3c). Since the dye-marker coloured the complete ductal system, more precise correlation of the two separate intraductal abnormalities was not possible.

In the last breast cancer patient (patient 9) the white light ductoscopy showed a possible red-coloured epithelial lesion with NCVs of 3.38 at 6 cm from the nipple. By autofluoresences mode, a clear epithelial lesion was seen (elevated NCV) which was microscopically identified as DCIS and lobular neoplasia were found outside the area of the ductoscopic lesion.

### Prophylactic patients

In none of the patients undergoing prophylactic mastectomy abnormalities were found with either white light or autofluorescence ductoscopy. This was confirmed by histology of the studied ducts, showing no abnormalities.

### Follow up

**Fig. 4:**
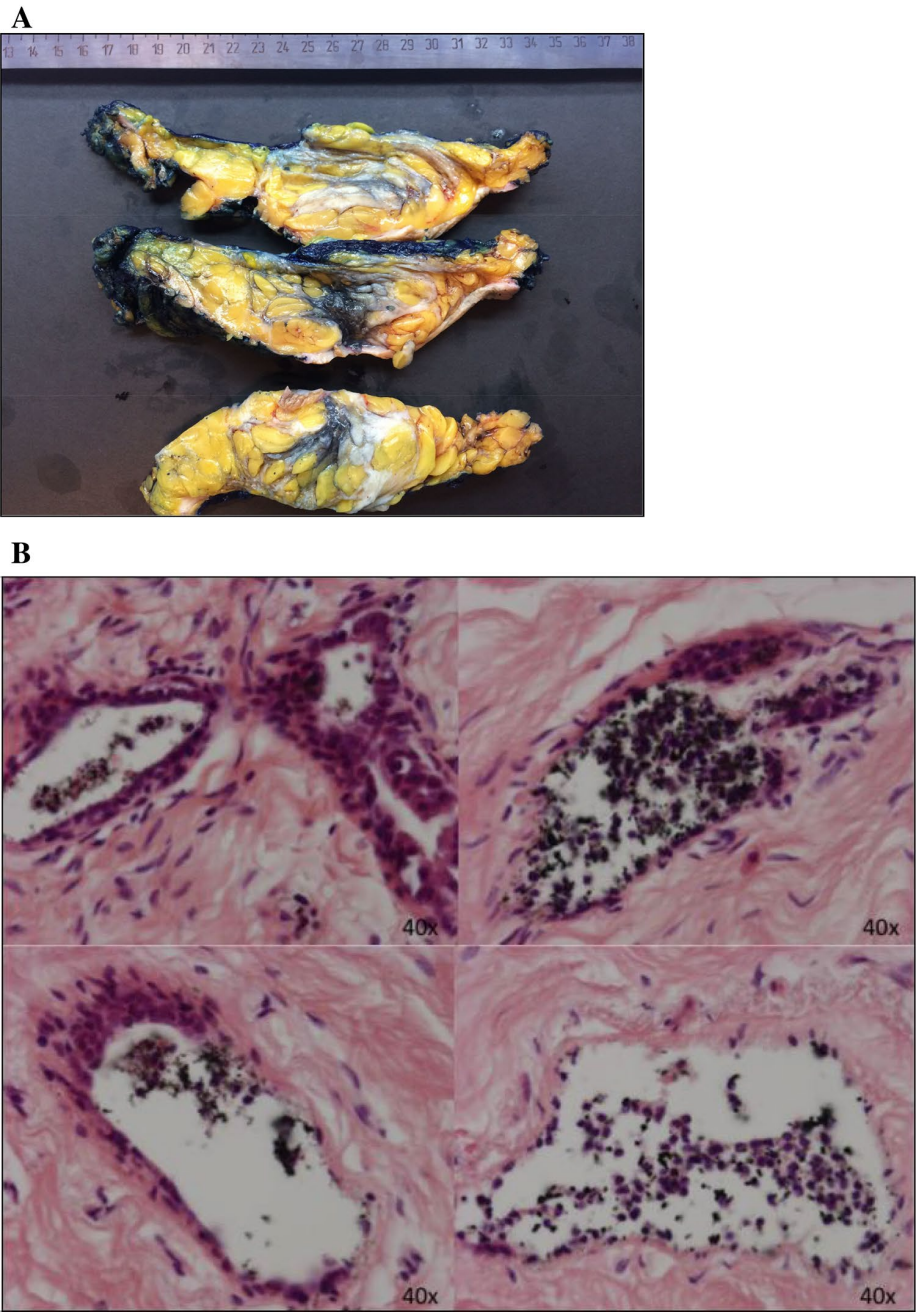
**a** Marking of the ductoscopic visualized duct in a patient undergoing prophylactic mastectomy for BRCA1. At (white light and autofluorescence) ductoscopy no abnormalities were found. Intraductal marking with Black Eye Endoscopic marker™ showed macroscopically clear localization for embedding. **b** Microscopically, the intraductal marking is easily traceable and the extravasation is not visible. The ductal marking shows the histological changes previously reported to be caused by ductoscopy: periductal clefts, epithelial detachment and epithelial loss [67]

Following ductoscopy, all patients underwent mastectomy. All resections were radical. Sentinel node biopsy was performed in all patients undergoing therapeutic surgery. In one patient the sentinel node contained a micro-metastasis. Adjuvant systemic therapy and radiation therapy was given in one patient. Median follow-up after surgery was 4 months (range 2–6). In all patients of the prophylactic cohort, immediate reconstruction was performed. In one patient undergoing prophylactic mastectomy with tissue expanders for subsequent reconstruction, bilateral necrosis of skin and nipple occurred, for which surgical necrotectomy was performed. No other complications occurred. Perforation of one or more ducts during ductoscopy occurred in five patients. Although this may limit endoscopic view (*n* = 1), it is without consequence for the patient. Subsequently inserted intraductal marker macroscopically showed extraductal diffusion from the ducts with perforations, without impairing microscopic localization (Fig. 4).

## Discussion

**Fig. 5:**
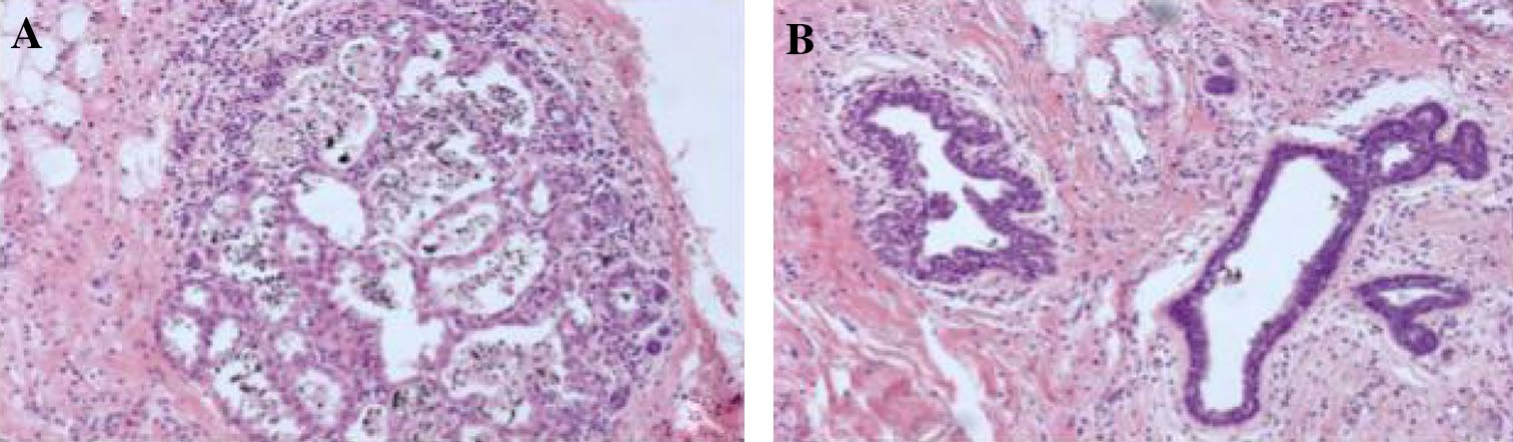
Histology of the examined ductal system of figure **a** and **b** show the intraductal black dye marking and apocrine metaplasia and ductal hyperplasia. Since the dye-marker colours the complete examined the ductal tree, precise correlation of the two separate intraductal abnormalities shown in **a** and **b** is not possible

This exploratory study indicates that autofluorescence ductoscopy is technically feasible in both breast cancer patients and high-risk women, with successful cannulation in 8 of 9 (89%) women. Autofluorescence ductoscopy was capable of identifying ductal hyperplasia and apocrine metaplasia and showed to be correctly negative in histological normal ducts. The used technique of intraductal marking with endoscopic dye-marker resulted in accurate macro- and microscopic localization of the studied duct (Fig. 5).

Although autofluorescence ductoscopy was positive in ductal hyperplasia and apocrine metaplasia, breast cancer precursor lesions were not found in this pilot study. The used autofluorescence settings are optimal for early-stage disease in other hollow organs such as the bronchus and gastro-intestinal tract, but further optimization of the imaging parameters may be necessary to increase specificity for premalignant lesions in the breast. Intraductal debris was seen in the lumen of the normal duct, which can be misinterpreted as cancer, appropriately appeared negative in autofluorescence mode, demonstrating the differentiating potential of autofluorescence ductoscopy in white light endoscopically suspect lesions. The continuous irrigation of saline solution not only ensured that the milk ducts remained open but also that the debris was washed away. Consequently, we did not encounter any interference with the image.

No lesions were found in women undergoing prophylactic mastectomy for high risk of breast cancer. These negative endoscopic findings were confirmed by histology. To evaluate the diagnostic accuracy of autofluorescence ductoscopy for the screening of high-risk women, a larger cohort in whom a number of premalignant lesions are present needs to be studied and prospectively followed with ductoscopy.

In previous studies using ductal lavage to guide risk-reducing strategies in high-risk women, (conventional) ductoscopy has already been suggested as a risk assessment tool for high-risk women [32, 33]. Danforth et al. compared ductoscopic findings with ductal lavage cytology in the contralateral high-risk breasts of breast cancer patients and visualised intraductal lesions in 83% of the ducts with atypical cytology [34]. On the contrary, in a study of ductal lavage cytology in asymptomatic, high-risk patients, poor concordance with histology was found and ductoscopy added little to this evaluation [35]. Although ductal lavage fails in yielding adequate specimens for reliable cytological diagnosis [36], diagnosis by the use of proteomic biomarkers in serum or methylation in nipple aspiration fluid or ductal lavage forms a promising alternative [37–41]. Methylation of specific genes is known as an early hallmark of carcinogenesis and can be detected in an only small amount of DNA, providing a potential method for early tumour detection. Addition of autofluorescence ductoscopy could possibly assist in visualising and locating early lesions. Together with the currently being studied novel intraductal treatment modalities e.g. via intraductal targeted therapy by RNA interference [42], intraductal chemotherapy [43] or intraductal laser ablation [44], this would form an appealing approach for early detection and treatment.

Some previous studies showed the success of ductoscopy in finding the intraductal lesion causing PND before or during duct excision [20, 21, 45]. In recent years, a biopsy tool was introduced that can be used through the working channel of the ductoscope enabling intraductal biopsy or removal of found lesions [18, 23, 46]. This so-called interventional ductoscopy has already been described before as a safe alternative for classic open surgery in patients with PND [18, 45, 46], but wider implementation requires further validation studies.

There are several more issues that need to be addressed. Most breast cancer arises from the terminal ductolobular unit (TDLU), where the ducts are narrow [47]. For ductoscopic examination of these TDLUs further minimization of diameter is needed. More challenging could be the anatomy of the breast, with the discrepancy between a number of duct and orifices in the nipple due to several ducts arising in the same cleft of the nipple [48]. Complete endoscopic examination will be difficult and sampling error could occur. Here, biomarker evaluation, e.g. RNA analysis of PND in ductal lavage or nipple aspiration fluid could be of additional value.

Also, the current techniques of ductoscopic diagnostic tissue acquisition are far from optimal. The ‘basket’-intervention device is only feasible in polypoid lesions [18, 23, 46, 49, 50] and intraductal biopsy devices are not commercially available [51, 52]. For histologic correlation of the endoscopic findings of this study were dependant of dye-marker injected through the working channel with subsequent surgical excision. This procedure caused marking of a complete single ductal system, precluding correlation of multiple lesions within one duct. Therefore, the development of a commercially available biopsy device suitable for superficial epithelial lesions remains much warranted.

Our results may have clinical implications for another patient group. Due to the association with breast cancer, numerous women with pathologic nipple discharge and negative imaging undergo exploratory surgery to rule out malignancy and to treat symptoms, despite breast cancer being found in a minor 3–7% in this patient group [18, 46, 53–56]. Ductoscopy has been used as a diagnostic modality to rule out malignancy, but while some studies reported a significant correlation [57, 58], others found no specific data except for gross morphological abnormalities such as papillomas [22, 59–61]. A recent network meta-analysis showed that white ductoscopy has a high specificity (98%) but low sensitivity (44%) for the detection of breast cancer in patients with pathological nipple discharge with no radiological suspicion for malignancy [62]. Since intraductal lesions (such as papillomas) can be removed with relative ease and histopathologically analysed [18, 46], autofluoresence might not be of added value in these cases. However, autofluorescence may help to increase the sensitivity of ductoscopy for the detection of breast cancer of lesions of the ductal wall, improving risk assessment and correlated treatment decisions.

It is estimated that 20–40% of BRCA1/2 patients who would never develop breast cancer are grossly overtreated with preventive mastectomies [2–6]. Stratification within these high-risk group remains elusive and more sensitive screening methods are warranted, although primary prevention with less radical treatment methods would be the ultimate solution. The current study shows that autofluorescence ductoscopy could be a feasible tool to stratify these high-risk groups. However, larger groups are necessary to determine the diagnostic performance of auto fluorescence ductoscopy. Nonetheless, we do expect auto fluorescence to be a valuable addition to ductoscopy for the detection of breast cancer precursor lesions since auto fluorescence has shown to be superior to white light endoscopies for the detection of precursor lesions in other cancer types of epithelial origin [63–66].

In conclusion, ductoscopy with the addition of autofluorescence is feasible in diagnosing intraductal breast lesions and could possibly increase specificity for endoscopic morphologically suspicious findings. However, this technique needs to be optimized and studied more intensively before it will be applicable in clinical practice.
